# A Step Towards Real-Time Release Testing of Pharmaceutical Tablets: Utilization of CIELAB Color Space

**DOI:** 10.3390/pharmaceutics17030311

**Published:** 2025-02-28

**Authors:** René Brands, Trieu Nam Le, Jens Bartsch, Markus Thommes

**Affiliations:** Laboratory of Solids Process Engineering, Department of Biochemical and Chemical Engineering, TU Dortmund University, 44227 Dortmund, Germany

**Keywords:** real-time release testing, in-line monitoring, UV/Vis spectroscopy, tableting, CIELAB color space, color parameters, porosity, tensile strength

## Abstract

**Background:** The pharmaceutical industry is shifting from end-product testing towards real-time release testing. This approach is based on the continuous collection of process data and product information, which is finally utilized for the release decision. For continuous direct compression, spectroscopic technologies are preferred due to their short acquisition time and non-destructive nature. **Methods:** Here, the feasibility of the CIELAB color space was demonstrated for porosity and tensile strength. Five different formulations were processed, varying in particle size and deformation behavior. The compression forces were varied from 3 to 18 kN and the CIELAB color space was measured in-line using a UV/Vis probe implemented in the ejection position of the tablet machine. **Results:** Increasing the main compression force during tableting decreases the tablet surface roughness and porosity. In addition, the tablet tensile strength increases. These changes affected the reflection behavior of radiation on the tablet surface, resulting in a change in the chroma value C*. These dependencies were utilized for the in-line monitoring of porosity and tensile strength. Linear relations were observed for all formulations as exemplary, indicated by sufficient coefficients of determination and verification runs. **Conclusions:** Finally, UV/Vis diffuse reflectance spectroscopy in combination with a CIELAB color space transformation was demonstrated to be a suitable real-time release tool.

## 1. Introduction

Pharmaceutical manufacturers are increasingly focusing on continuous processes rather than batch production to improve the drug product quality and to reduce the manufacturing cost as well as the equipment footprint. Furthermore, continuous processing enables a comparably easier scale-up in terms of process time [1,2,3,4]. In this context, tableting is particularly interesting, as tablets hold a high market share among oral solid dosage forms [5]. However, to achieve the required process understanding and control to operate a tableting process in a continuous fashion, the implementation of Process Analytical Technologies (PAT) is crucial [6]. Particularly suitable in this context are in-line methods, as these provide real-time data about the process and product quality. These data can be utilized for the real-time release testing (RTRT) of the drug product. This includes the continuous monitoring and evaluation of critical quality attributes (CQAs) throughout the manufacturing process to ensure product quality and compliance [7] and replace the traditional end-product testing [8,9].

Typical CQAs for a tablet amongst others are the active pharmaceutical ingredient (API) content, porosity, and tensile strength. These are therefore an integral part of real-time release testing strategies [7]. While the first attribute is set by the material pre-blend, the physical properties refer to the applied compression force for a given tablet weight. However, spectroscopic methods have been proven to be particularly advantageous for the in-line determination of the mentioned CQAs. These methods achieve high measurement frequencies and are usually non-destructive by nature [10,11,12].

Near-infrared spectroscopy (NIR) and Raman spectroscopy were implemented for the monitoring of the chemical composition of tablets [13]. This enabled the monitoring of API and excipient contents [14,15]. However, multivariate data analysis is required in combination with statistical models [16]. In contrast, the API content in tablets could be also monitored in-line based on univariate analysis using UV/Vis spectroscopy [17].

Various spectroscopic methods were implemented to determine the porosity of tablets, like in-line NIR. However, a multivariate data analysis in combination with statistical models was required [18,19]. The application of terahertz spectroscopy enabled the measurement of a large number of physical tablet parameters. These included the at-line measurement of tablet porosity. Furthermore, a simultaneous determination of tablet height and porosity was possible by using terahertz time-domain spectroscopy [20,21]. However, no in-line implementation of this technology in a rotary tablet press without a sampling device was possible to date [22]. Furthermore, off-line acoustic emission spectroscopy was utilized to measure the porosity of tablets [23].

Tablet hardness was off-line monitored simultaneously with tablet porosity using NIR [24]. The application of NIR spatially resolved spectroscopy enabled the off-line detection of differences in tablet hardness and API content. Furthermore, off-line ultrasound transmission measurements were performed to determine the hardness of tablets [25,26]. Gas in scattering media absorption spectroscopy in combination with photon time of flight spectroscopy were utilized for off-line tablet hardness and optical porosity measurements [27].

Accordingly, existing methods for determining the physical properties of tablets involve either multivariate data analysis or are performed with off-line testing. At the same time, UV/Vis spectroscopy was successfully implemented into a rotary tablet press for API content in-line monitoring using univariate data analysis and is characterized by remarkably short measurement times [28].

**Figure 1:**
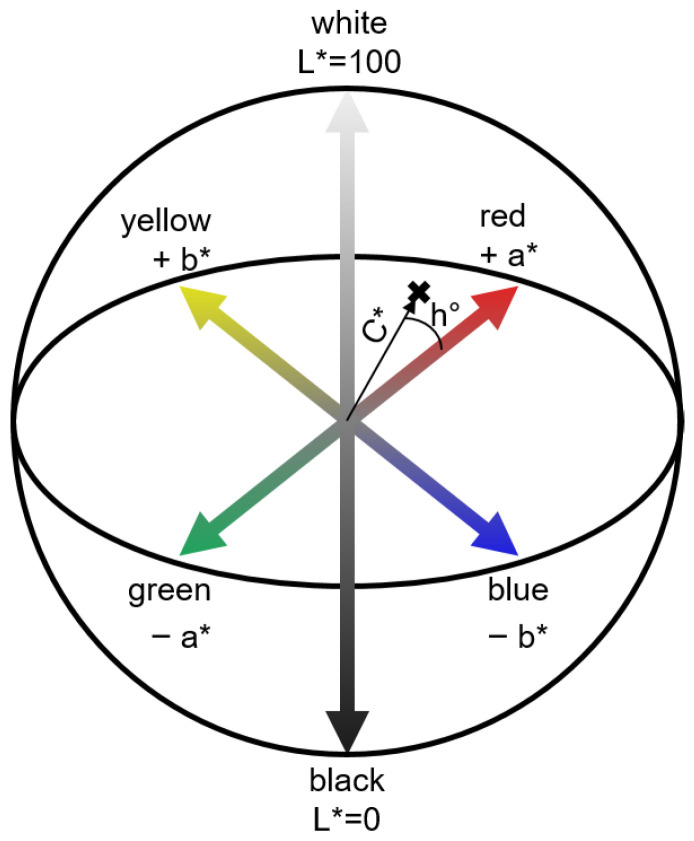
Graphical representation of the CIELAB color space with polar coordinates C* and *h°*.

In order to determine the physical properties of the tablet, the raw spectra were converted into the CIELAB color space (Figure 1), developed by the International Commission on Illumination (CIE). Hereby, the Vis range from 380 to 780 nm is utilized. This color space describes all visible colors to the human eye in a three-dimensional cartesian coordinate system using the L*, a*, and b* parameters [29]. The L* value corresponds to the lightness that is equivalent to human perception in a range from 0 (black) to 100 (white). The a* and b* values describe different color ratios, whereby the a* value represents the ratio of green to red and the b* value the ratio of yellow to blue. These parameters can be converted to the polar coordinates C* and *h°*. Here, the chroma value C* describes the color saturation and hue is described by *h°*. Based on off-line studies, a correlation between the color parameters and the density of compressed powders was already identified [30].

**Figure 2:**
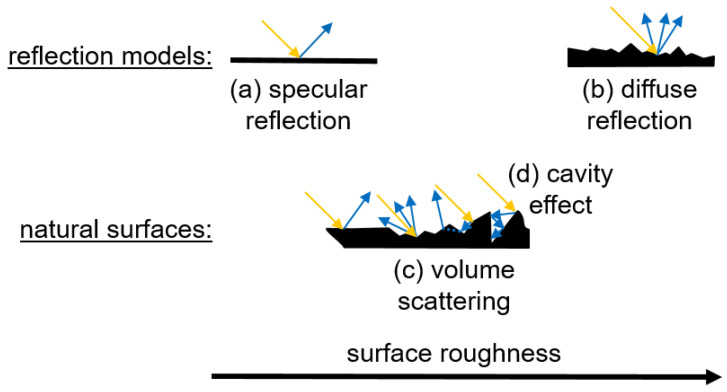
Schematic diagram of radiation interaction with a smooth surface (**a**), a rough surface (**b**), and natural surfaces including volume scattering (**c**) and cavity effect (**d**). Adapted according to [31].

In this context, the reflection depends on the surface properties. If the radiation hits a smooth surface, specular reflection occurs to a greater extent. Hereby, the angle of incidence is identical to the angle of reflection, which leads to a coplanar reflection (Figure 2) [31,32]. In contrast, the radiation is scattered in all directions on a rough surface, which is referred to as diffuse reflection [33]. Volume scattering can occur in the presence of fine particles on the surface, which leads to the transmission of the radiation through the fine particles [34]. Also, cavities in the surface can trap the radiation and reduce reflection, known as the cavity effect [35]. On real surfaces, such as tablets, several types of reflection occur simultaneously, depending on surface roughness and porosity.

Therefore, the aim of this study was to extend the UV-VIS spectroscopy approach to a simultaneous real-time monitoring of chemical and physical tablet properties by applying the CIELAB color space. For this purpose, a probe was implemented in a rotary tablet press and five formulations were pressed with main compression forces from 3 to 18 kN in order to alter the physical properties like porosity and tensile strength systematically. The composition, deformation behavior, and particle sizes were varied. First, the corresponding experimental investigations considered a proof-of-concept test. Then, the influence of tablet characteristics such as surface roughness on the determination signal was investigated and a validation was carried out with a particular focus on linearity and range. Finally, the feasibility of the developed method for in-line monitoring and real-time release testing was demonstrated.

## 2. Material and Methods

### 2.1. Materials and Formulations

Five different tablet formulations were chosen based on physical properties e.g., particle size, deformation behavior, and the ability to absorb radiation (Table 1).

Two different α-lactose monohydrate qualities were exploited to investigate the influence of particle size on diffuse reflection and the C* value. The lactose quality Foremost 310 (Foremost Farms USA, Baraboo, WI, USA) utilized for the formulation L_fine_ and Tablettose 80 (Meggle, Wasserburg am Inn, Germany) used for the formulation L_coarse_. Emcocel 90M (JRS Pharma, Rosenberg, Germany) was chosen as microcrystalline cellulose. Moreover, theophylline monohydrate (Thermo Fisher Scientific, Waltham, MA, USA) was added to investigate the influence of absorbing substances, while magnesium stearate (Ligamed MF-2-V, Peter Greven, Bad Münstereifel, Germany) served as a lubricant. In this study, theophylline monohydrate was used as a model drug due to its plastic compression behavior, which makes it suitable for tableting, and because of its UV/Vis radiation absorption characteristics.

### 2.2. Methods

#### 2.2.1. Particle Size Characterization

The size distributions of all the used materials were determined with a laser diffraction system (Mastersizer 3000, Malvern Panalytical, Malvern, UK) equipped with a dispersion unit (Aero S, Malvern Panalytical, Malvern, UK). All the measurements were carried out in triplicate.

#### 2.2.2. Heckel Analysis

A Heckel analysis was performed in order to determine the influence of deformation behavior. Therefore, tablets were pressed on an eccentric tablet press (EK 0, Korsch AG, Berlin, Germany) with 10 kN compression force and data were evaluated according to Heckel [36]. Then, the plasticity refers to the yield stress 1/K.

#### 2.2.3. Tableting

All the materials corresponding to the formulation, except the lubricant, were blended for 12 min in a 3D shaker mixer (Turbula, WillyA. Bachofen AG, Muttenz, Switzerland) at 32 rpm in a 20 L blending vessel. Each batch consisted of 4 kg of powder. In a subsequent step, the lubricant was added and a second blending was performed for 1.5 min.

Tableting experiments were executed on a rotary tablet press (102i, Fette Compacting, Schwarzenbek, Germany) for six different main compression forces varying from 3 to 18 kN in equidistant levels, namely 3, 6, 9, 12, 15, and 18 kN. Here, the tablet press was equipped with 24 pairs of punches (8 mm diameter, EU 19) and a three-chamber feed frame (FOM, Fette Compacting, Schwarzenbek, Germany) with round spokes paddle wheels. The tablet press turret speed was set to 13.9 rpm and the feed frame speed to 90 rpm. Tablet weight was set to 320 mg. Corresponding filling curves were selected depending on the bulk density of the formulation.

#### 2.2.4. Surface Roughness

The surface topography was determined using a non-contact profilometer (Profilometer MicroProf, FRT GmbH, Gladbach, Germany). Areas of 2 × 3 mm^2^ on the tablet sidewall, which corresponds to the sample area of the UV/Vis probe, were examined at a measuring frequency of 100 Hz. Three tablets were measured for each main compression force. The surface roughness was calculated based on the elevation (*z*(*x*,*y*)) of the tablet at the tablet surface (*S*). Therefore, the height component attributed to tablet curvature was removed from the total elevation resulting in an elevation based on surface roughness with a mean value close to zero. The surface arithmetic mean height (Sa) calculated (Equation (1)) based on absolute (positive) elevation values gives a common parameter for surface roughness [37].

(1) $$Sa=\frac{1}{S}\iint \left|z(x,y)\right|dxdy$$

#### 2.2.5. Porosity

Porosities (*ε*) were calculated according to European Pharmacopoeia 11 (Chapter 2.9.23). Therefore, tablet volumes were determined based on the geometric dimensions, taking into account the curvature of the tablet. The tablet density ($\rho_{t}$) obtained by volume and weight measurements (ST50, Sotax, Aesch, Switzerland) was related to the solid density ($\rho_{s}$) determined by helium pycnometry (Ultrapyc 1200e, Anton Paar, Graz, Switzerland) (Equation (2)) [38].

(2) $$\varepsilon =\left(1-\frac{\rho_{t}}{\rho_{s}}\right)*100$$

#### 2.2.6. Tensile Strength

For each compression force, representative samples of 20 tablets were randomly selected and the diameter (d), tablet thickness (h), central cylinder thickness (t), and breaking force (F) were determined using a semi-automatic tablet testing system (ST50, Sotax, Aesch, Switzerland). The hardness in terms of tensile strength ($\sigma_{TS}$) (Equation (3)) was calculated according to the United States Pharmacopoeia 35 (Chapter 1217) using the extended version for convex surface tablets [39].

(3) $$\sigma_{TS}=\left(\frac{10F}{\pi d^{2}}\right){\left[\left(\frac{2.84h}{d}\right)-\left(\frac{0.126h}{t}\right)+\left(\frac{3.15t}{d}\right)+0.01\right]}^{-1}$$

#### 2.2.7. Porosity and Tensile Strength In-Line Monitoring

**Figure 3:**
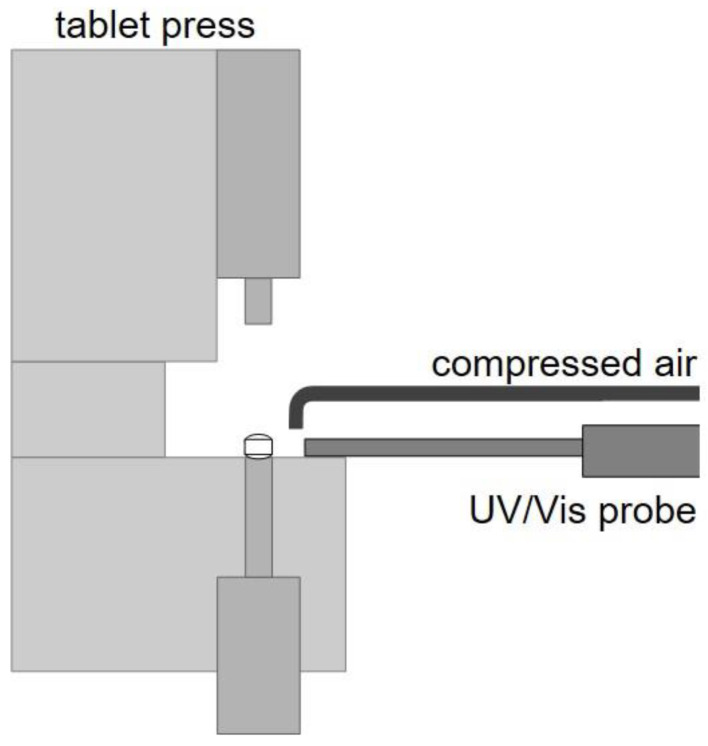
UV/Vis probe implemented in rotary tablet press including compressed air stream.

The in-line monitoring of the physical tablet properties was performed with the combination of a UV/Vis spectrophotometer (Inspectro X, ColVisTec AG, Berlin, Germany) with a modified reflectance polymer melt probe (RPMP, CoVisTec AG, Berlin, Germany). Herein, the glass fibers are arranged in a 6-1 array and the six outer glass fibers emit radiation from 224 to 820 nm, while the inner glass fiber guides the reflected radiation back to the sensor. The probe is implemented in a horizontal alignment in the tablet press to expose tablets on the tablet sidewall that are currently being ejected and still remain on the lower punch (Figure 3) [17]. Here, the distance from the probe to the tablet was set to 4 mm. Furthermore, a gas jet and a vacuum unit have been implemented to keep the front of the glass fibers free of particles. The UV/Vis probe emits 35 light flashes of 2 ms each per measurement. Combined with the necessary integration time, this results in a measuring frequency of 1.5 Hz. By selecting these measurement parameters, only one tablet is exposed per measurement.

#### 2.2.8. Conversion of Spectral UV/Vis Data into CIELAB Color Space

The D65 10° standard illuminant was utilized to express the CIELAB color space relative to a white reference, which corresponds to the average midday light in Europe. For this purpose, the spectrum of the visible range was multiplied by that of the standard illuminant D65 10°, and the resulting curve was multiplied by the CIE standard observers. These correspond to the three color-matching functions according to the average response for human visible wavelengths. Based on this, the *X*, *Y*, and *Z* standard color coordinates were calculated by using numerical integration. Afterwards, these coordinates were utilized to calculate the coordinates L*, a*, and b* according to Equations (4)–(7) [40].

(4) $$L^{*}=116f\left(\frac{Y}{Y_{n}}\right)-16$$

(5) $$a^{*}=500\left[f\left(\frac{X}{X_{n}}\right)-f\left(\frac{Y}{Y_{n}}\right)\right]$$

(6) $$b^{*}=200\left[f\left(\frac{Y}{Y_{n}}\right)-f\left(\frac{Z}{Z_{n}}\right)\right]$$

(7) $$f\left(x\right)=\left\{\frac{x^{\frac{1}{3}}\;\;\;\;\;\;for\;x>{\left(\frac{24}{116}\right)}^{3}}{\left(\frac{841}{108}\right)x+\left(\frac{16}{116}\right)\;\;for\;x\leq {\left(\frac{24}{116}\right)}^{3}}\right\}$$

Here, *X_n_*, *Y_n_*, and *Z_n_* are reference white values for CIE standard illuminant D65. Finally, the polar coordinate C* (chroma, relative saturation) was calculated by using Equation (8) [40]:

(8) $$C^{*}=\sqrt{a^{*2}+b^{*2}}$$

Thus, the CIELAB color space was constructed (Figure 1). The C* value, also known as the color saturation, was used in the following for the in-line monitoring of the tablet properties porosity and tensile strength.

In addition, the CIELAB color space was used to exclude tablets where tablet position and radiation exposure were not in phase during the measurement. This step is necessary since the tablet press is not synchronized with the UV/Vis probe. For this purpose, the L* value of the CIELAB color space was selected as a threshold.

## 3. Results

### 3.1. Raw Material and Formulation Characterization

Particle size tests indicate the lowest particle size for Foremost 310, the main component of the L_fine_ formulation (Table 2). In contrast, Tablettose 80 has a 1.6 times larger particle size and is the main component of the formulation L_coarse_. Emcocel 90M has a similar particle size to Tablettose 80. The largest particle size was found for Theophylline and the smallest for the lubricant magnesium stearate.

The results of the Heckel analyses indicate a difference between formulations based on lactose and those based on microcrystalline cellulose in terms of yield stress 1/K (Table 3). Thereby, the microcrystalline cellulose formulations seem to exhibit a more plastic deformation behavior compared to the formulations with lactose, which is consistent with the literature [41].

### 3.2. CIELAB Color Space and Surface Roughness Effects of Compression Force

The tablets consisting of the different formulations were produced with different main compression forces from 3 up to 18 kN on the rotary tablet press, and, simultaneously, the UV/Vis reflectance spectra were monitored in-line. Based on this, the C* value of the CIELAB color space was calculated.

**Figure 4:**
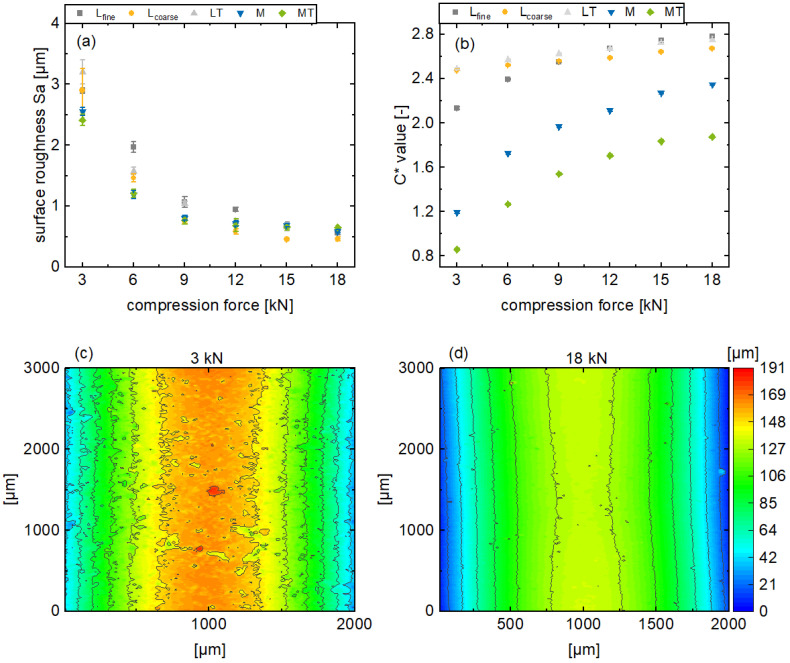
Presentation of the tablet surface roughness *Sa* (**a**) and the in-line monitored C* values (**b**) for various main compression forces for the formulations L_fine_, L_coarse_, LT, M, and MT (av ± CI, n_C*_ = 147–198, *n_Sa_* = 3) and exemplary results from a profilometer measurement of the tablet sidewall for tablets based on formulation L_fine_ (**c**,**d**).

Generally, the surface roughness, in terms of *Sa*, decreases for all the formulations as the main compression force increases, until it converges to a minimum surface roughness of approximately 0.5 µm (Figure 4a). Additionally, this effect of the main compression force on the surface appearance is also observable in Figure 4c,d. With an increasing main compression force, the surface irregularities of the tablets decrease and the surface appears smoother. Further results regarding the remaining formulations can be found in the Supplementary Materials. However, the surface roughness of the plastic formulations M and MT decreases relatively less in this main compression range compared to the brittle formulations. Furthermore, the surface roughness of the plastic formulations converges towards a minimum at a main compression force of 9 kN, while this effect is only noticeable for brittle formulations at higher main compression forces.

In contrast to the surface roughness, the color saturation increases with a higher main compression force for all the formulations (Figure 4b). Moreover, the C* values for the rather brittle formulations (L_fine_, L_coarse_, LT) tend to be higher than those of the plastic formulations (M, MT). In addition, the plastic formulations are more affected by the variation of the compression force with respect to the slope. The described differences regarding the color saturation of the tablet surfaces refer to the corresponding topography. If mechanical stress is applied to brittle particles during tableting, the particles break into smaller fragments. For plastic particles, on the other hand, deformation takes place. Here, the resulting topography tends to be smoother with less irregularities expressed by a lower Sa value. However, both mechanisms are generally promoted for higher compression forces leading to less surface roughness, which has an influence on the reflection of radiation. When a parallel beam of radiation is incident on a rough surface, the angles of incidence of the radiations are different and the radiation is scattered omnidirectional. This referred to as diffuse reflection. If more radiation is scattered, correspondingly less radiation is returned, the observed surface appears less color-intensive, and the color saturation (C* value) decreases.

In addition, it can be observed that the C* value increases with decreasing surface roughness. This is due to the previously described dependency between surface roughness and light scattering. Nevertheless, it can be observed that the color saturation changes even with constant surface roughness at high main compression forces, which needs further elaboration.

### 3.3. Porosity In-Line Monitoring by Using the C* Values

The surface roughness considers the near-surface porosity, which directly influences the surface topography via the pores, structural characteristics, and irregularities that are inherent to this property. Consequently, there is a reasonable expectation of a correlation between the C* value and tablet porosity.

**Figure 5:**
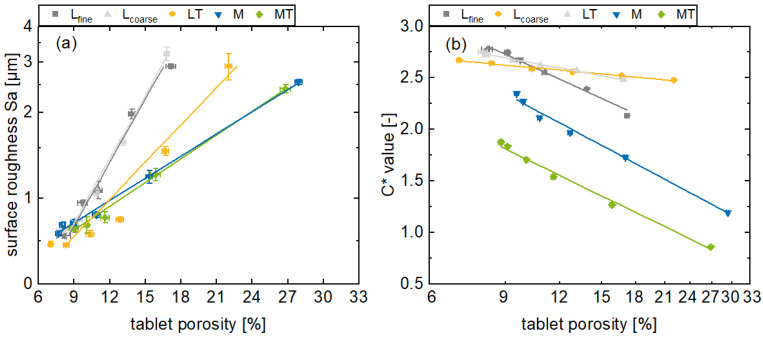
Presentation of the surface roughness Sa (**a**) and the in-line monitored C* values (**b**) for the formulations L_fine_, L_coarse_, LT, M, and MT with different porosities (av ± CI, n_C*_ = 147–198, *n_porosity_* = 20).

Porosity and surface roughness as well as porosity and C* value are correlated in a first approximation by linear functions for a logarithmic representation. However, there are systematic deviations that indicate a non-linear relationship. Nevertheless, this correlation is suitable for the particular range under consideration in this study, indicated by a coefficient of determinations of 0.95 for the L_coarse_ formulation up to 0.99 for the M formulation (Figure 5a). These results are comparable to off-line NIR and Raman measurements, where coefficient of determination values equal to or higher than 0.9 were achieved [18,24]. The incoming radiation can be directed into the pores and reflected there in terms of cavity scattering and thus reduce the color saturation. Likewise, the radiation can pass through the material from pore to pore and thus be scattered in terms of volume scattering, decreasing the radiation intensity and reducing the color saturation [31]. Furthermore, deep penetration of the radiation is possible with high porosity. Tortuosity is another important factor in this context. With decreasing porosity, these effects decrease relatively and the light scattering is reduced, leading to an increased color saturation due to increased specular reflection.

With respect to the results of the experimental investigations in this context (Figure 5b), the tablet porosity decreases with increasing main compression force for all the formulations as expected, since the free volume in the tablet decreases with an increasing main compression force. However, the porosities at 3 kN are higher for the plastic formulations compared to the brittle formulations, as brittle materials tend to form smaller fragments during tableting under mechanical stress and fill the free pore spaces (Figure 5). The formulation L_coarse_ with an increased d_50_ exhibits a higher initial porosity compared to the formulation L_fine_, given that larger gaps remain between the particles when they are pressed to tablets. These gaps contribute to the porosity, as these are not completely filled with particles. The in-line monitored C* values and the off-line determined porosities indicate a linear correlation when the porosity is transformed to a logarithmic scale. Here, the color saturation increases for all the formulations, while the porosity decreases. This can be confirmed by the sufficient coefficients of determination, and is consistent with the literature, where a correlation between color saturation and density of materials was observed [30]. For the brittle formulations, the coefficients of determination are L_fine_ 0.982, L_coarse_ 0.990, and LT 0.989. On the other hand, for the plastic formulations, the M and MT are at 0.993 and 0.993. Thus, the plastic formulations show a comparatively higher degree of correlation. Nevertheless, the results are all sufficient for the use as a real-time release tool application considering the linear correlation between the C* value and tablet porosity in the respective range.

### 3.4. Tensile Strength In-Line Monitoring by Using the C* Value

**Figure 6:**
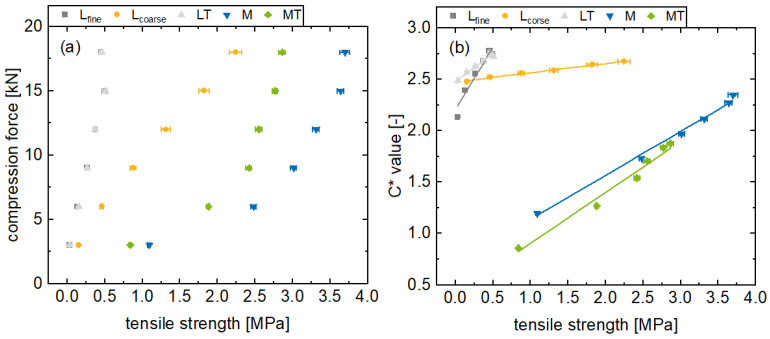
Presentation of the compression forces (**a**) and the in-line monitored C* values (**b**) for the formulations L_fine_, L_coarse_, LT, M, and MT with different tensile strengths (av ± CI, n_C*_ = 147–198, n_tensile strength_ = 20).

Reducing the porosity leads to denser particle packing. The reduced distance between the particles increases the van der Waals forces and leads to an increase in tablet tensile strength [42]. This can be observed for all the formulations, whereby the tablet tensile strength increases with a higher main compression force (Figure 6a). Generally, a linear correlation between the main compression force and tensile strength can be recognized for brittle formulations, whereby an exponential correlation for plastic formulations was found. The plastic formulations M and MT lead to harder tablets in general with tensile strengths of up to over 3 MPa. Furthermore, the L_coarse_ formulation exhibit significantly higher tensile strength values at similar main compression forces, compared to L_fine_. This difference in tensile strength can be attributed to the manufacturing process of the L_coarse_ lactose quality and the corresponding particle size distribution. Presumably, structural failure in the tablets at 18 kN main compression force is recognizable for the L_fine_ and LT formulations. Thereby, this structural failure leads potentially to a decreased tensile strength at an 18 kN main compression force compared to 15 kN. Accordingly, an increased main compression force did not lead to harder tablets, but more likely exceeded stresses in the tablet, which led to weak spots. These findings are consistent with comparable data in the literature [43].

Increasing the tensile strength through higher compression forces leads to increased C* values (Figure 6b) in general. Within the range considered in this study, the deviation from linear behavior is negligible. In this respect, correlations can be recognized, indicated by the high coefficients of determination of L_fine_ 0.944, LT 0.985, L_coarse_ 0.991, M 0.995, and MT 0.982. However, the two formulations with structural failure exhibit lower coefficients of determination. The outcomes of this study demonstrate a high degree of similarity to previously reported off-line NIR and Raman measurements. In these cases, coefficient of determination values greater than or equal to 0.9 were reported for off-line hardness measurements [18,24]. Finally, the correlation between color saturation and tablet tensile strength can be attributed to the previously described influence of porosity on the surface structure and scattering behavior, whereby porosity correlates with tablet tensile strength. However, effects such as structural failure inside the tablet do not seem to have an influence on the surface roughness and surface porosity and therefore do not influence the reflection behavior. Therefore, this effect is not evident for the method. Nevertheless, it should be noted that this in-line monitoring is intended for the real-time release of tablets in a manufacturing process. The process will be designed in such a way that there will be no structural errors. For such processes, main compression forces of 18 kN are uncommon and accordingly this finding is not intended to be limiting. Consequently, the tensile strength of tablets in terms of tensile strength can be monitored in-line with the C* value.

### 3.5. Real-Time Monitoring Case Study

The suitability for in-line monitoring in a rotary tablet press of the physical tablet properties was demonstrated exemplarily for formulation M. For this purpose, tablets were produced continuously for 32 min. The main compression force was adjusted every 8 min in a random order (4, 17, 10.5, and 7 kN), while the single tablet properties were determined via the UV/Vis probe in real-time. Simultaneously, samples were taken every 2 min, and the tablets were tested off-line with respect to porosity and tensile strength using a semi-automatic tablet testing system.

**Figure 7:**
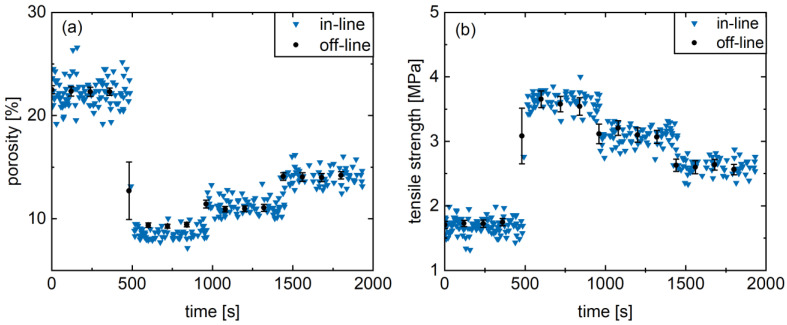
Presentation of the off-line as well as in-line results of the calibration model verification for porosity (**a**) and tensile strength (**b**) of the formulation M. (av ± CI).

Generally, a high level of agreement between in-line and off-line values can be observed (Figure 7), highlighting the feasibility of the developed UV/Vis method for the determined range of tablet porosity and tensile strength. In addition, the measuring frequency is higher with the in-line determination and the tensile strength of the tablets is measured in a non-destructive manner. Hereby, a distinction between the different levels and the resulting physical properties is feasible. This indicates a high precision for the applied compaction force variations with respect to the natural process fluctuations. Consequently, after a prior calibration porosity and tablet tensile strength can be predicted in real-time via in-line UV/Vis spectroscopy using the C* value of the CIELAB color space.

## 4. Conclusions

In this study, a UV/Vis spectroscopy fiber probe was implemented in the ejection position of a rotary tablet press and successfully utilized for the in-line monitoring of physical tablet properties. The tablets were produced with different main compression forces. Here, particularly tablet porosity and tablet hardness in terms of tensile strength were investigated. Additionally, the tablets surface roughness was characterized using a laser profilometer.

The Vis region of the spectra was transformed into the CIELAB color space. Subsequently, the color saturation C* (chroma) was calculated and correlated to the tablet porosity and tensile strength. Here, increasing the main compression force leads to higher color saturations in terms of C*. These changes are related to the tablet surface structure which affects the reflection behavior of radiation on the tablet surface. Reduced surface irregularities lead thereby to reduced omnidirectional scattering and thus increase the color saturation of the tablets.

Based on this knowledge, calibration models were successfully developed for tablet porosity and tablet tensile strength. This includes five different formulations, which differ in particle size, deformation behavior, and chemical composition. These formulation characteristics were identified as influencing factors for the C* value, with plastic formulations exhibiting higher sensitivity. Additionally, the suitability for real-time monitoring was proven based on a case study for formulation M with a high degree of agreement between off-line and in-line methods.

In conclusion, UV/Vis spectroscopy is a promising tool for a real-time release tool in tableting, based entirely on mechanical models and capturing both the chemical composition and physical properties of the tablets such as porosity and tensile strength.

## Figures and Tables

**Table 1:** List of formulation compositions.

Name	Substance	Weight Fraction [wt%]
L_fine_	Foremost 310	99.5
	Ligamed MF-2-V	0.5
L_coarse_	Tablettose 80	99.5
	Ligamed MF-2-V	0.5
LT	Foremost 310	89.5
	Theophylline	10
	Ligamed MF-2-V	0.5
M	Emcocel 90M	99.5
	Ligamed MF-2-V	0.5
MT	Emcocel 90M	89.5
	Theophylline	10
	Ligamed MF-2-V	0.5

**Table 2:** Particle size analysis results with corresponding d_10_, d_50_, and d_90_. (av ± s; *n* = 3).

Substance	d_10_ [µm]	d_50_ [µm]	d_90_ [µm]
Foremost 310	14.37 ± 0.17	67.60 ± 0.46	158.60 ± 2.16
Tablettose 80	28.80 ± 0.08	109.33 ± 0.46	283.33 ± 4.76
Emcocel 90M	32.33 ± 0.19	109.67 ± 0.47	234.33 ± 2.06
Theophylline	48.63 ± 5.13	115.00 ± 1.73	211.00 ± 9.54
Ligamed MF-2-V	2.12 ± 0.02	6.87 ± 0.12	34.60 ± 2.43

**Table 3:** Heckel analysis results including yield stress 1/K. (av ± s; *n* = 3).

Formulation	1/K [MPa]
L_fine_	412.21 ± 24.32
L_coarse_	368.17 ± 5.40
LT	406.01 ± 17.36
M	218.23 ± 3.57
MT	230.20 ± 9.38

## Data Availability

Data is contained within the article.

## Supplementary Materials

The following supporting information can be downloaded at: https://www.mdpi.com/article/10.3390/pharmaceutics17030311/s1, Figure S1: Surface roughness L_coarse_; Figure S2: Surface roughness LT; Figure S3: Surface roughness M; Figure S4: Surface roughness MT.

## Abbreviations

area	*S*	mm^2^
breaking force	*F*	N
blue–yellow value	*b**	/
central cylinder thickness	*t*	m
chroma	*C**	/
diameter	*d*	m
elevation	*z*(*x*,*y*)	µm
green–red value	*a**	/
hue	*h°*	/
lightness	*L**	/
porosity	*ε*	%
solid density	$\rho_{s}$	g/cm^3^
standard color coordinates	*X*, *Y*, *Z*	/
surface roughness	*Sa*	µm
tablet density	$\rho_{t}$	g/cm^3^
tablet thickness	*h*	m
tensile strength	*σ*	MPa
